# Editorial: Biology and treatment of high-risk CLL

**DOI:** 10.3389/fonc.2022.1109950

**Published:** 2023-02-20

**Authors:** Eugen Tausch, Jitka Malcikova, John C. Riches, Jennifer Edelmann

**Affiliations:** ^1^ Division of Chronic Lymphocytic Leukemia (CLL), Department of Internal Medicine III, Ulm University, Ulm, Germany; ^2^ Center of Molecular Medicine, Central European Institute of Technology, Masaryk University, Brno, Czechia; ^3^ Department of Internal Medicine – Hematology and Oncology, University Hospital Brno, Masaryk University, Brno, Czechia; ^4^ Centre for Haemato-Oncology, Barts Cancer Institute, Queen Mary University of London, London, United Kingdom; ^5^ Department of Haemato-Oncology, Barts Health NHS Trust, St. Bartholomew’s Hospital, London, United Kingdom; ^6^ Ulm University Hospital, Ulm, Germany

**Keywords:** chronic lymphocytic leukemia (CLL), high-risk, TP53, targeted therapy, omics, registries

In the era of chemoimmunotherapy, high-risk chronic lymphocytic leukaemia (CLL) was defined by the presence of *TP53* loss and/or *TP53* mutation and by refractoriness to purine‐analogue based treatment (no remission or remission under 6 months in duration), respectively (1). The advent of chemo‐free treatment regimens at all disease stages requires a re‐definition of the term “high‐risk CLL”, but this constitutes a challenging task when considering the rapidly evolving treatment landscape and the increasing knowledge about CLL pathobiology obtained over recent years. While CLL characterization used to focus on clinical parameters and limited genomic analyses (2), samples can nowadays be analysed in a far more comprehensive manner, since “omics” technologies allow an integrative analysis of data obtained at the genomic, epigenomic, transcriptomic, and proteomic level as well as an assessment of spatial tumor heterogeneity and tumor evolution over time. Next to intrinsic CLL characteristics, a growing understanding about the interplay of CLL cells with their microenvironment provides additional aspects to consider for CLL risk stratification. The wealth of information that can in principle be obtained for each CLL case challenges the identification of biomarkers conferring poor prognosis and predicting treatment failure.

From a clinical perspective, high-risk CLL may hitherto best be defined by refractoriness, non‐durable response or intolerance towards the two drug classes that have become most relevant for CLL treatment: covalent BTK inhibitors (BTKi) such as ibrutinib and acalabrutinib and BCL2 inhibitors (BCL2i) such as venetoclax (3). However, this clinical definition entails the exhaustion of two treatment lines by the time that high‐risk patients become identifiable, a situation in which the current drug approval status has only a limited number of alternative drug classes on offer. To adapt and personalize CLL therapeutic approaches in a way that enables durable eradication of all CLL clones in high‐risk patients in first‐line, it will be essential to define biomarkers identifying respective patients before or early after treatment initiation.

Validating the prognostic or predictive impact of potential biomarkers has been complicated by a growing number of targeted compounds, immunotherapeutic agents and products for adoptive cell therapy that need to be tested in prospective clinical trials. The plethora of options for new monotherapies and combination therapies to be evaluated requires large numbers of CLL patients to be enrolled on clinical trials. The distribution of patients across a multitude of therapeutic options makes it difficult to reach the statistical power necessary to validate the impact of each potential biomarker for each individual treatment approach. In keeping with this notion, not even the prognostic impact of *TP53* disruption has yet been conclusively clarified in the setting of BTKi and BCL2i based treatment. This clearly illustrates how difficult it will be to develop multivariate prognostic models that take genetic, epigenetic, transcriptional, phenotypic and clinical parameters into account and permit an individualised treatment recommendation for every CLL patient that considers clinical benefit as well as costs.

The scope of the Research Topic “Biology and Treatment of High‐risk CLL” is to provide a state-of-the-art overview of the pathobiological mechanisms underlying high‐risk CLL, to list and critically evaluate biomarkers that have been associated with a high‐risk disease character and to outline the implications of these biomarkers on response to different treatment approaches including chemoimmunotherapy, targeted therapy and cellular immunotherapy. Another issue discussed within the Research Topic is Richter transformation, which is defined as the development of a high‐grade lymphoma in patients with a previous or concurrent diagnosis of CLL or small lymphocytic lymphoma (SLL) and frequently associated with a dismal prognosis.

The Research Topic is opened by a review article in which Kwok and Wu. discuss prognostically relevant biologic alterations in CLL observed at the genetic, transcriptional, epigenetic and microenvironmental level. In case respective data was available, alterations are regarded as dynamic processes throughout which the tumor can evolve towards therapeutic resistance and progression. Building up on this, the authors create a future vision how multidimensional tumor heterogeneity and tumor growth dynamics could become parameters in a prognostic model allowing more personalised treatment choices.

After this introductory review, three articles focus on *TP53* alteration serving as a defining criterion of high‐risk CLL and as established predictor for chemoresistance (4). Soussi and Baliakas. first illuminate the pathobiology of *TP53* alterations in CLL as compared to other malignancies and show how to utilize *TP53* locus‐specific mutation databases and cancer genome databases for a correct interpretation of *TP53* variants. Lazarian et al. then summarize available data on low‐burden *TP53* mutations defined by a variant allele fraction <10% and discuss the open research question as to what extent low‐burden *TP53* mutations are clinically relevant. In a Brief Research Report, Catherwood et al. present data on *TP53* alterations obtained from a real-world cohort of 2332 CLL cases analysed by next generation sequencing and fluorescence *in situ* hybridization (FISH).

In the next section of the Research Topic, three additional poor prognostic biomarkers identified at the genetic level are highlighted. First, Chatzikonstantinou et al. present available data on the prognostic and potentially predictive value of karyotype complexity in CLL and discuss the impact of low, intermediate and high genomic complexity against the background of concomitant high-risk biologic features. Subsequently, Zavacka and Plevova. shed light on chromothripsis defined as a genomic event by which a single chromosome or a limited number of chromosomes are shattered into pieces and reassembled in an error-prone process. The authors discuss the potentially underlying biological causes for this event and review the impact of chromothripsis on CLL disease progression and treatment response. Then, Nguyen-Khac. addresses the role of *MYC* rearrangements in CLL with a focus on concurrent loss of the *TP53* gene locus, a constellation termed “double‐hit CLL”. This latter topic is backed up by an original research article by Ondrouskova et al. describing the frequency of *MYC*‐rearranged CLL in a cohort of 303 cases from a single center and dissecting the types of the genomic alteration responsible for *MYC* rearrangement in an extended cohort.

The Research Topic then focusses on two cell signaling pathways shown to influence prognosis. First, B‐cell receptor signaling is highlighted as a key survival pathway for CLL cells. Gerousi et al. explain the concept of B‐cell receptor stereotypy in CLL and summarise data on CLL subsets defined by stereotyped B‐cell receptors and associated with an aggressive clinical course. In a complementary perspective article, Nicolo et al. put emphasis on the fact that alterations in the B‐cell receptor immunoglobulin light chain can also drive the development of high‐risk CLL. Afterwards, Edelmann. elucidates the impact of *NOTCH1* mutations and further alterations associated with de-regulated NOTCH1 signaling.

Richter transformation is a dreaded complication of CLL evolution with an aggressive clinical course and no established treatment options. Condoluci and Rossi. address the issue of Richter transformation by giving insight into its pathobiology and summarizing data on potential treatment options recently evaluated in clinical trials.

The last section of the Research Topic is dedicated to treatment of high‐risk CLL. To systematically approach the question how high‐risk CLL behaves under various treatment approaches, Straten et al. select *TP53* alterations, Del(11q), genomic complexity, unmutated IGHV mutation status, stereotyped B‐cell receptor subsets, *NOTCH1* mutation and *BIRC3* mutation from the list of poor prognostic factors in CLL and discuss their clinical impact based on available data from clinical trials testing chemotherapy, chemoimmunotherapy, anti‐CD20 treatment, BTK inhibition, BCL2 inhibition, Pi3K inhibition and/or allogeneic stem cell transplantation. The authors also comment on the next generation kinase inhibitors zanubrutinib, pirtobrutinib and duvelisib, the anti‐CD20 monoclonal antibody ublituximab and on CAR-T-cell and CAR‐NK cell therapeutic approaches. To explain the role of allogeneic stem cell transplantation and CAR T‐cell therapy in more detail, Barbanti et al. add a review article focussing on the use of these cellular approaches for the treatment of high‐risk CLL and Richter transformation.

In a concluding Opinion Article, Edelmann, Malcikova et Riches propose a new definition of high‐risk CLL taking disease intrinsic risk factors as well as additional elements with an influence on overall survival into account.

## Author contributions

All authors made a substantial, direct and intellectual contribution to the conceptual design of the Research Topic. JE wrote the Editorial. All authors contributed to the article and approved the submitted version. Eugen Tausch, Jitka Malcikova and John Riches contributed equally to the conceptual design of the Research Topic." in Author contributions.
